# HPV vaccination in Kenya: a study protocol to assess stakeholders' perspectives on implementation drivers of HPV vaccination and the acceptability of the reduced dose strategy among providers

**DOI:** 10.3389/frhs.2023.1233923

**Published:** 2023-08-02

**Authors:** Grace Umutesi, Lynda Oluoch, Bryan J. Weiner, Elizabeth Bukusi, Maricianah Onono, Betty Njoroge, Lucy Mecca, Kenneth Ngure, Nelly R. Mugo, Ruanne V. Barnabas

**Affiliations:** ^1^ Department of Global Health, University of Washington, Seattle, WA, United States; ^2^ Center for Clinical Research (CCR), Kenya Medical Research Institute, Nairobi, Kenya; ^3^ Division of National Vaccines and Immunization Program, Ministry of Health, Nairobi, Kenya; ^4^ School of Public Health, Jomo Kenyatta University of Agriculture and Technology, Nairobi, Kenya; ^5^ Division of Infectious Diseases, Massachusetts General Hospital, Boston, MA, United States; ^6^ Departement of Medicine, Harvard Medical School, Boston, MA, United States

**Keywords:** cervical cancer prevention, human papillomavirus vaccine, Kenya, consolidated framework for implementation research, theoretical domain framework, theoretical framework for acceptability, implementation science

## Abstract

**Background:**

Cervical cancer is the leading cause of cancer-related deaths among Kenyan women. Persistent infection with high-risk oncogenic Human papillomavirus (HPV) genotypes is a necessary cause of cervical cancer. HPV vaccines are safe, durable, and efficacious in preventing incident HPV infections. In Kenya, despite efforts to increase HPV vaccination, coverage remains low. We sought to assess: (1) barriers and facilitators of HPV vaccination from the perspective of adolescent girls and young women (AGYW), their guardians as well as stakeholders involved in HPV vaccine delivery, and (2) the acceptability of the single dose of the HPV vaccination among healthcare providers (HCPs).

**Methods:**

Our study is nested within the KENya Single-dose HPV-vaccine Efficacy study (KEN SHE) that sought to test the efficacy of single-dose bivalent (HPV 16/18) and single-dose nonavalent (HPV 16/18/31/33/45/52/58/6/11) vaccination. We are conducting this study in Kiambu, Nairobi, and Kisumu counties. In these counties, we are interviewing stakeholders (*n* = ∼25), selected based on their role in HPV vaccination at the county and national levels. Interviews are audio recorded and conducted in English or Swahili. The semi-structured interview guides were designed based on: (1) the Theoretical Domains Framework (TDF) for AGYW and guardians and (2) the Consolidated Framework for Implementation Research (CFIR) for other stakeholders. The Theoretical Framework of Acceptability (TFA) was leveraged to design the survey administered to HCPs (*n* = ∼309) involved in HPV vaccination. We will develop a codebook based on emerging codes from the transcripts and constructs from the TDF and CFIR. Emerging themes will be summarized highlighting similarities and differences between and within the different stakeholder groups and counties. Descriptive statistics and a *χ*^2^ test will be used to assess the distribution of responses between the different sites and regression analysis will be used to assess factors associated with high acceptability of the single-dose strategy while controlling for confounding variables.

**Discussion:**

Our study will describe key barriers and facilitators that affect HPV vaccination from the perspective of multiple stakeholders as well as insights on the perspective of HCPs towards the single-dose strategy to inform the designing of strategies to increase HPV vaccination uptake in Kenya and comparable settings.

## Introduction

Cervical cancer is the fourth leading cause of death among women (1). In 2020, the burden of new cases and deaths due to cervical cancer was concentrated in low-and-middle-income countries (LMIC), accounting for 90% of the global cancer incidence and mortalities (2). Sub-Saharan Africa bears a high prevalence of cervical cancer with a mortality rate of 94.1 per 100,000 in 2018 (2, 3). Invasive cervical cancer (ICC) is one of the few cancers with a known infectious etiology, and persistent infection with high-risk oncogenic human papillomavirus (HPV) is a necessary cause for ICC (4). However, HPV vaccines are safe, durable, and efficacious in preventing incident HPV infections that lead to cervical cancer (5, 6). The World Health Organization (WHO)'s Global Cervical Cancer Elimination Strategy has three pillars, one of which is achieving 90% HPV vaccination coverage for age-eligible girls. Unfortunately, the global HPV vaccination coverage for age-eligible adolescent girls remains low where it was estimated at 15% in 2019 (5, 7, 8).

In Kenya, cervical cancer is the leading cause of cancer-related deaths among women, resulting in approximately 3,400 deaths annually (9). In accordance with the recommendations from the WHO, a two-dose schedule for HPV vaccination was introduced in Kenya in 2019 targeting 10-year-old adolescent girls through facility-based delivery (10). At the time of the HPV vaccination launch, social mobilization, and community education efforts were conducted to raise awareness and ignite vaccine uptake (11, 12). Since the national introduction, the HPV vaccination coverage in Kenya has been suboptimal where 33% of eligible AGYW received the first doses in 2020 and this estimate increased to 77% in 2021 but only 31% of targeted AGYW had received 2 doses of the HPV vaccine in 2021 (9, 13). In Kenya, HPV vaccination coverage has been adversely impacted by delivery processes and vaccine hesitancy among healthcare providers (HCPs) and at the community level. Additionally, the global COVID-19 pandemic increased vaccine hesitancy at the community level, the lack of confidence among HCPs sparked their reluctance to promote HPV vaccination, and the lack of community engagement and education after the initial launch of the program resulted in knowledge gaps that fueled HPV vaccination refusals (11, 12, 14, 15).

These implementation-related challenges highlight the need for comprehensive evidence from stakeholders on factors that facilitate or impede the delivery and uptake of HPV vaccination. The overall objective of this study is to understand why HPV vaccination coverage remains low for AGYW in Kenya, despite evidence endorsing the crucial role of HPV vaccination in cervical cancer prevention. This study aims to generate knowledge on implementation drivers of HPV vaccination in Kiambu, Nairobi, and Kisumu County. Our specific aims are to (1) assess barriers and facilitators to HPV vaccination delivery in the three counties, and (2) assess the acceptability of the single dose of HPV vaccination among HCPs. Findings from this study will be shared with the Kenyan Ministry of Health and they will potentially contribute to informing the design of the national guideline for HPV vaccination and generate evidence for decision-makers.

## Methods and analysis

This study is nested within the KENya Single-dose HPV-vaccine Efficacy study (KEN SHE, ClinicalTrials.gov number NCT03675256). The KEN SHE study is a randomized, multicenter, double-blind, three-arm, controlled trial that sought to test the efficacy of single-dose bivalent (HPV 16/18) and single-dose nonavalent (HPV 16/18/31/33/45/52/58/6/11) HPV vaccination compared with meningococcal vaccine among Kenyan women of 15–20 years of age (16). Interim data analysis done at 18 months of the KEN SHE study showed that the HPV vaccines were highly effective with a vaccine efficacy of 97.5% to prevent incident persistent HPV 16/18 infection.

### Aim 1: assessing barriers and facilitators to HPV vaccination delivery in three Kenyan counties

#### Study design and study population

We are leveraging a qualitative study design to assess barriers and facilitators to HPV vaccination delivery. Study participants are stakeholders involved in HPV vaccination delivery program at the national, county, sub-county, and community levels. Study participants are being selected based on their position and role in the delivery of HPV vaccination for AGYW in Kenya. They include:

(1) From the central government: staff from the National Immunization Program and the Reproductive Health Division at the Ministry of Health (MOH); staff from the Ministry of Education (MOE); national implementing partners (NGOs, advocacy groups, etc.)
(2) From the county level: the health minister or coordinator at county level, heads of county hospitals, other local implementing partners.
(3) From the delivery level: heads of hospitals where HPV vaccines are delivered, the nurse in charge of immunization at the health facility; healthcare frontline vaccine providers at facilities; clinical or medical officers where applicable; principals and teachers as well as opinion leaders (including religious leaders and area chiefs).

Adolescents, girls, and young women (AGYW) aged 10 years and older who were vaccinated and those who were not vaccinated along with their guardians, are also being interviewed to capture details on implementation drivers and barriers from the AGYW and guardians' perspective.

#### Sample size determination

Study participants are purposefully sampled based on their involvement in the delivery of HPV vaccination. Eligible participants are enrolled and interviewed until saturation is reached for each participant category. We estimate that in each of the three counties, we will interview ∼5 participants from each category and 10 from the national level, however, more participants are being recruited and interviewed when saturation is not reached after interviewing the estimated number of participants.

#### Recruitment procedures

The KEN SHE study team has engaged the Ministry of Health, Ministry of Education, and other relevant implementers at the national and county levels to recruit relevant study participants for interviews. Identification of eligible key informants is done in liaison with county managers and the target group includes teachers, religious leaders, and healthcare providers. Additionally, a list of key informants specific to each county is being supplemented with a list of AGYW and guardians (both vaccinated and unvaccinated). AGYW aged 10 years and older are being recruited from the parent study, health facilities, the community, schools, and colleges. The identification of unvaccinated AGYW is done by community health workers (CHWs) with experience in research recruitment, and who have been involved in the parent study, the KEN SHE trial. Parents or guardians approached for permission before speaking to AGYW and their consent is sought before interviewing AGYW under the age of 18. Informed assents are being obtained for all study participants before enrolment. All interviews are being held in an environment that is convenient for participants. This includes clinics for providers, offices for implementers and decision makers, churches, schools, or other community settings for opinion leaders, AGYW, and guardians.

### Data analysis and management

#### Data collection tools

To select constructs relevant to this study, we mapped out each construct from the Theoretical Domain Framework (TDF) and the Consolidated Framework for Implementation Research (CFIR) to the KEN SHE study setting (17–19). Constructs that matched the study setting and local context were leveraged to design the semi-structured interview guide to capture information on the selected TDF and CFIR constructs. We used the TDF to design the interview guide for AGYWs and their guardians. This approach ensured that the interview guide accounted for the characteristics of the implementation environment and determinants of behavior (Supplementary Appendix I). Additionally, the CFIR served as a guide to design the interview guide for opinion leaders and decision-makers since CFIR focuses more on aspects of the health system and it is more appropriate in contexts where the individual domain or determinants of behavior are less relevant (Supplementary Appendix II). This interview guide was also adapted from a CFIR-guided tool that was used in Mozambique for similar purposes (20). Both interview guides were pilot tested and refined prior to conducting the interviews with targeted study participants. Domains and constructs captured in each interview guide as well as sample questions are illustrated in Table 1.

**Table 1:** Example of questions from the interview guides for AGYW, parents, opinion leaders and decision makers.

Framework	Domain	Construct	Example of questions
Theorical Domain Framework (TDF)^a^	Intervention	Relative advantage	Do you feel HPV vaccine is better than the existing system of screening for and identifying cervical cancer in women in Kenya?
		Adaptability	Are there alterations that should be implemented to improve uptake of HPV vaccine? If yes, which ones
		Cost	What is the cost that you have incurred for you/your daughter to receive a full HPV vaccination?
	Outer setting	Cosmopolitanism	Is there networking happening between AGYW/their parents regarding HPV vaccine? or with AGYW/parents from other settings?
		Peer Pressure	To what extent have other AGYW in your setting received the HPV vaccine?
		External Policy & Incentives	What kind of financial or other incentives influenced your decision to receive/allow your daughter to receive the HPV vaccine?
	Implementation context (Inner Setting)	Structural Characteristics	Where do most adolescents receive the HPV vaccine in your community? How is their experience in general?
		Implementation climate	To what extent does the implementation of HPV vaccination take a backseat to other high-priority initiatives going on now in your community?
		Readiness for implementation	What kind of support or actions are needed to make HPV vaccine delivery successful in your community/setting?
	Characteristics of individuals	Knowledge and Belief about HPV vaccine	How do you feel about the HPV vaccine being delivered to AGYW in your setting?
		Self-efficacy	How confident are you that you/your community will be able to use the intervention (agree to receive HPV vaccine)?
	Process	Planning	How was planning for implementation of the HPV vaccination carried out in your community?
		Engaging	Who are the key people who have been engaged in the HPV vaccine delivery in your community/setting?
	The health system and policy context	Health systems context	In your opinion, what knowledge and beliefs about the HPV vaccine impacted implementation? At the community and individual level?
		Policies and guidelines	Are there any policies, programs, or guidelines that might affect the delivery and uptake of HPV vaccines in your community/setting?
Consolidated Framework for Implementation Research (CFIR)^b^	Intervention characteristics	Evidence strength and quality	To what extent were you provided with or made aware of the evidence supporting the introduction of the HPV vaccine?
		Adaptability	In your opinion, what alterations should be implemented to improve uptake of HPV vaccine?
		Design Quality & Packaging	How is public health education on the importance of the HPV vaccine undertaken?
		Cost	Are you aware of the estimated cost of implementing HPV vaccination in government health facilities/your area?
	Outer Setting	AGYW Needs & Resources	In your opinion, to what extent are implementers aware of the needs and preferences of AGYWs?
		Cosmopolitanism	Is there networking happening between the Kenyan MOH and other MOHs during the HPV roll out? or with other county managers?
		External Policy & Incentives	What kind of international policies, mandates have influenced the decision to roll out the HPV vaccine in Kenya/your setting?
	Implementation context (Inner Setting)	Network & Communications	What was the structure of the network of stakeholders for HPV delivery in Kenya/your setting?
		Implementation climate	[Compatibility sub-construct] How well does the HPV vaccination fit with existing work processes and practices in Kenya/your setting?
		Readiness for implementation	[Leadership Engagement sub-construct] What has been the level of support from leaders?
	Process	Planning	How was planning for implementation of the HPV vaccination carried out at your facility, district, county, nationally?
		Engaging	Who are the key people who have been engaged in the HPV vaccine delivery in your setting/nationally?
		Executing	Has the HPV vaccination delivery been implemented according to the implementation plan?
		Reflecting and evaluating	How has the national rollout of HPV vaccination been monitored and evaluated?
	The health system and policy context	Health systems context	How do you think the rollout of the HPV vaccination is going in your area/community/the health system?
		Policies and guidelines	Are there any policies, programs, or guidelines that might affect the delivery and uptake of HPV vaccines?

^a^ Leveraged to interview AGYW and guardians.

^b^ Leveraged to capture insights from opinion leaders and decision makers.

#### Data analysis

We will use constructs of the TDF and the adapted CFIR to guide the analysis of interview transcripts. A codebook will be developed based on emerging codes from the transcripts and the interview guide developed using TDF and CFIR. Once consensus on the codebook has been reached; the remaining interviews will be coded, and appropriate measures will be used to ensure that the coding approach is reliable. Regular coding checking will be performed to ensure that the coding strategy used is reliable. Field notes will be used to inform the interpretation of findings and all coding work will be done with Atlas.ti version 9. Participants' demographics will be summarized in a table detailing the distribution of key characteristics of each targeted group, the number of participants interviewed in total, and for each group. Emerging codes will be used to identify key themes that will be categorized within constructs and domains of the appropriate framework. Themes that do not fit within constructs and domains of the TDF or CFIR will also be listed as new insights that emerged from the interviews. We will summarize similarities and differences of key themes between and within groups.

### Aim 2: assess the acceptability of the single dose of HPV vaccination among healthcare providers

#### Study design and study population

We are using a concurrent mixed-methods study design to assess the acceptability of the single-dose strategy among healthcare providers. Study participants are healthcare providers involved in the HPV vaccination delivery at different levels of the health system. This includes nurses, clinical officers, pharmacists, pharmacy technicians, medical officers, and other relevant healthcare providers.

#### Sample size determination

Based on the assumption that each health facility has at least one healthcare provider responsible for HPV vaccination with a total of 1,568 health facilities in the three counties (Kiambu: 364, Nairobi ∼1,000, Kisumu ∼200), we estimated the total number of healthcare providers responsible for HPV vaccination to be ∼1,568. Assuming that 50% of healthcare providers in the three countries are involved in HPV vaccination, at least 309 health healthcare providers will need to complete the 10-item survey to assess the acceptability of the reduced dose strategy among providers in the three counties with a 95% confidence interval that the real value is within a ± 5% of the survey results (Table 2).

**Table 2:** Summary of study participants for each aim.

Study aim	Study participant category	Number of participants	Comment
Aim 1 (KIIs)	AGYWs	15	∼5/county
	Parents/Guardians	15	∼5/county
	Community leaders/Opinion leaders	15	∼5/county
	Healthcare providers	15	∼5/county
	Ministry of Health/Education (National & County) and Implementing partners	10	
Aim 2	Healthcare providers	309	• ∼100/county • Including 15 from aim 1
**Total**		**364**	

#### Recruitment procedures

The study coordinator collaborated with County Immunization Managers to map health facilities where providers are being recruited to participate in a survey that assesses their acceptability of the single-dose strategy. To minimize selection bias and obtain diverse perspectives, the survey link is being shared among providers at different levels of the healthcare system and *via* healthcare providers' WhatsApp groups to capture insights from providers at various levels of the health system, including private, public, and missionary health facilities, as well as different administrative levels such as county, sub-county, and health center.

### Data analysis and management

#### Data collection tools

We used the theoretical framework for acceptability (TFA) to design the survey focusing on questions that are relevant to Kenya and the KEN SHE study sites (21). Survey responses include qualitative data from free-text responses to the survey and quantitative data from selected responses to the survey.

#### Data analysis

We will employ constructs of the TFA to guide the analysis of text responses from the survey. A codebook will be developed inductively based on emerging codes from these responses and deductively from the TFA. Once consensus on the codebook has been reached, all text responses from the survey will be coded, and appropriate measures will be used to ensure that the coding approach is reliable. Participant demographics will be summarized in a table detailing the distribution of key characteristics, similar codes will be merged into key themes and categorized into domains of the TFA where applicable. Themes that do not fit within constructs and domains of the TFA will also be listed as new insights that emerged from the survey. All qualitative analyses will be conducted in Atlas.ti version 9. Additionally, descriptive statistics and a *χ*^2^ test will be used to assess the distribution of survey responses between the different sites. A multinomial logistic regression analysis will be used to assess the factors associated with the acceptability of the single-dose among healthcare providers controlling for confounding variables. All quantitative analyses will be performed in R-4.3.1. A joint display approach will be leveraged to mix qualitative and quantitative results to facilitate a mixed-method interpretation of findings.

#### Confidentiality and data storage

Trained study staff are conducting all study procedures in private and protecting the privacy and confidentiality of study participants. Study-related information are being stored securely at the study clinic. Study records that contain names or other personal identifiers, such as the informed consent forms, are being maintained separately and securely with limited access. Forms, lists, and any other listings that link participant numbers to identifying information are being secured in a separate locked file area. Data collection and administrative forms are being identified only by coded numbers and kept secure, with access limited to authorized study staff. Audio-recorded interviews and interview transcripts are labeled with a study ID-specific site for each site and study participant (e.g.,: NBO/HCW/KI001—Nairobi/Healthcare worker/key informant#1) and stored securely. All study databases are being protected with password access systems and all datasets including interview transcripts and the survey responses will be stored in a password-protected SharePoint folder managed by the KEMRI team.

#### Informed consent processes

Informed written consents are being sought and obtained from all study participants and parents or guardians where applicable. For illiterate study participants, the consent form is being read out loud for them and their signature or thumbprint is being obtained for those who agree to participate in the study. All study participation is strictly voluntary, and participants can refuse study participation at any time.

All participants will go through an informed consent process and assent is being obtained from parents or guardians for all minor AGYW. All materials used in providing informed consent, including consent forms, were reviewed, and approved by the ethics committees.

## Discussion

Achieving WHO cervical cancer elimination goals requires understanding context-specific bottlenecks, factors that contribute to increasing HPV vaccination coverages, and uptake to protect as many women as possible from HPV infections and future cervical cancer incidences. Findings from this study will generate insights on barriers and facilitators that affect HPV vaccination from the perspective of a diversity of stakeholders at different levels of the health system (providers, opinion leaders, funders, implementers, AGYWs, and their parents) in Kenya. A variation of factors is expected between and within settings and other key characteristics of participants (gender, location of residence, level of training, role in the community, etc). Additionally, the acceptability of the single-dose strategy will vary depending on the providers' awareness of existing evidence on this new recommended vaccination schedule, their location, and level within the healthcare system. We do not expect the acceptability to vary among providers based on their demographic characteristics or education.

Insights from this study will contribute evidence to support the Kenya HPV vaccination delivery and uptake. Additionally, these findings will contribute to the development of setting-specific outreach, educational and training materials to disseminate evidence among different stakeholders which will contribute to increasing the HPV vaccination coverage in Kenya.

## Dissemination policy

The study team is committed to public dissemination of results of the formative research to participants, local stakeholders and policy makers in Kenya, the global scientific community. Dissemination of study results will follow principles of good participatory practice. Results will be published in conference abstracts and peer-reviewed journals. Study results will be disseminated through presentations to local stakeholders and policymakers in Kenya, including the Ministry of Health.
