# Supplementary material for: HPV vaccination in Kenya: a study protocol to assess stakeholders' perspectives on implementation drivers of HPV vaccination and the acceptability of the reduced dose strategy among providers
Source: Front Health Serv. 2023 Aug 2;3:1233923. doi: 10.3389/frhs.2023.1233923 (PMC10433907; doi:10.3389/frhs.2023.1233923)
Supplement: Supplementary file 1 [file Datasheet1.pdf]

AIM 1: AGYW & PARENTS IN DEPTH INTERVIEW GUIDE

Instructions:

- This in-depth interview (IDI) is intended to obtain information from AGYWs
- The objective of the IDI is to elicit views and insights from AGYW & their guardians about factors that facilitate or impede their ability to get the HPV vaccine.
- The interviewer should use, and adapt, if necessary, the lines of inquiry below to ask participants':
    - Knowledge on HPV vaccine
    - Their perceptions on HPV vaccine
    - Their sense of comfort with AGYW receiving HPV vaccine
    - Beliefs and assumptions
    - How easy it was for the AGYW/their parent to agree to HPV vaccine
    - What held the AGYW/parent back from agreeing that the AGYW receives the vaccine
    - Their perceptions of the service delivery environment
    - Their interpersonal experiences with facility staff and providers who provided the vaccine

- This interview should be conducted in a language that the AGYW/ their parent or guardian prefers, please confirm which language they prefer before starting

Introduction/Opening Statement:

[READ ALOUD – INTERVIEWERS CAN PARAPHRASE]:
Thank you for taking the time to participate in this in-depth interview on the factors that may facilitate or hinder the uptake of HPV vaccine among AGYW in Kenya.

The Kenya Ministry of Health (MoH) recommendations for HPV vaccination is a two-dose schedule 0 and 6 months similar to the World Health Organization Recommendations. Kenya currently has a national HPV vaccine program based on evidence with school-based vaccination for 9-10 years which was initiated in 2019. GAVI has supported two HPV vaccination demonstration projects from 2013-2015 and 2016-present in Kitui county (outside of our proposed study areas). Both demonstration projects used the quadrivalent HPV 16/18/6/11 vaccine in a school-based strategy that targeted girls aged 9-10 years, and the first achieved 85% coverage.
We aim to understand the key barriers and facilitators currently faced in the roll out of the HPV vaccination program. We are conducting this study to generate insights which will inform the MOH process for implementation. We are interested in your perceptions of HPV vaccine acceptability, the factors that you think may help or hinder health care workers and teams tasked with implementing HPV vaccine provision effectively and make it a sustainable component of cervical cancer prevention among AGYW in our communities.

AIM 1: AGYW & PARENTS IN DEPTH INTERVIEW GUIDE

This interview will take approximately 2h . With your permission, I would like to record the interview on a digital recorder [show the participant the digital recorder].
The recording will be kept securely and confidentially, and only used for the research purposes as described in the informed consent form [show the participant the form, which s/he has already signed].
Can you please confirm which language you would like us to use during this session?

Do I have your permission to record this session?

Thank you

INTERVIEWER SHOULD CONFIRM WITH THE KEY INFORMANT THAT S/HE HAS PERMISSION TO RECORD THE IDI ON A RECORDER. IF RESPONDENT CONSENTS, THE INTERVIEWER CAN START RECORDING NOW. RECORD THE TIME AT WHICH THE INTERVIEW BEGINS.

START TIME OF IDI: ________________________

Name of the interviewer:<br>____<br>Date of interview:<br>____<br>ID of Key Informant:<br>Eg:NBO/AGYW/KI001 - Nairobi/ AGYW/ key informant#1<br>____<br>Date on which informant received HPV vaccine:<br>____<br>Community and county were informant lives:<br>____<br>[For parents/ guardians] Number of children that the participant has:<br>____<br>Highest level of schooling of participant:<br>____<br>Location of the interview (e.g., participant's home, name of healthcare facility):<br>____<br>Age of the KII:
Marital status (single, married, divorced, widowed):

AIM 1: AGYW & PARENTS IN DEPTH INTERVIEW GUIDE

Duration of the interview (minutes):

Interview:

0. Vaccination status

i)Before we begin, would you mind telling me if you have received your HPV vaccine?

ii)How about your eligible daughter(s)?

iii)If yes, how many doses have you/ your daughters received?

1. The Intervention

Interviewer should ask about the AGYW/their guardians' views and experiences with respect to the following aspects of HPV vaccine. As the interviewer listens, s/he should make note of barriers and facilitators that the AGYW mentions.

1.a. Evidence strength and quality

i) To what extent were you provided with or made aware of the evidence on HPV vaccine?

ii)Where did you learn about the HPV vaccine?

ii) What do you and other AGYW/ parents think of the HPV vaccine?

iv)What evidence is needed on the HPV vaccine for AGYW/ parents to increase its uptake?

Probe

- ➔ *Quality concerns?*
- ➔ *Validity of evidence?*

1.b. Relative advantage

i) Could you tell me more about the HPV vaccine? In your opinion, what would you say are the benefits and risks of receiving HPV vaccine?

PROBE

ii))Do you feel HPV vaccine is better than the existing system of screening for and identifying cervical cancer in women in Kenya?

ii) What is better HPV vaccination or cervical cancer screening?

Probe

- ➔ *Knowledge of the AGYW/parent on the benefits of the HPV vaccine?*
- ➔ *Any perceived drawbacks of HPV vaccination from the AGYW/parent's perspective?*
- ➔ *Anyone they know with cervical cancer?*
- ➔ *Advantage of implementing HPV vaccination vs other CC preventive strategies*

1.c. Adaptability

i) In your opinion, what are factors that contribute to the uptake of HPV vaccine in your area among AGYW?
ii) In your opinion, what factors impede the uptake of HPV vaccination in your area among AGYW?
iii)Are there alterations that should be implemented to improve uptake of HPV vaccine? If yes, which ones
iv)Are there components that should not be altered?
v) What is the situation around HPV vaccination in your community?

Probe

- ➔ *What are the needed implementation adjustments and adaptations to meet local needs?*

1.d. Complexity

i)How complicated is it to receive the HPV vaccine in your setting?

PROBE

- ➔ *complicated in terms of uptake*
- ➔ *Complicated in terms of steps required to receive or complete the doses*

1.e. Design Quality & Packaging

i) What did you think about the way in which information on HPV vaccination has been presented to AGYW/ parents in your setting/community?
ii) How does available materials on HPV vaccination affect the uptake in your setting/ community?
iii)How is public health education on the importance of the HPV vaccine undertaken?

Probe

- ➔ Was the public educated on the vaccine before its introduction into health facilities?
- ➔ How long was the public educated on the vaccine before its introduction into health facilities
- ➔ How were community gatekeepers approached prior to the introduction of the HPV vaccine in health facilities?
- ➔ How is the continued community engagement post introduction of the vaccine being implemented

1.f. Cost [Might be more appropriate for parents/ guardians]

(i) What is the cost that you have incurred for you/ your daughter to receive a full HPV vaccination?

ii) What do you think is the cost that is incurred in your community by other AGYW/ their parents for them to be fully vaccinated?
iii) In your opinion, how should these resource needs/ cost barriers be addressed?

Probe

- *Percentage of the income spent to make sure the HPV vaccine is received (eg; transport, work day missed, need for caregiver for other children, etc.)*

2. Outer Setting

2.a. AGYW/ Parents' Needs & Resources

i) In your opinion, to what extent do you think HPV vaccination staff are aware of the needs and preferences of AGYW in your setting?
ii)To what extent have AGYWs' needs been taken into consideration by the HPV vaccination program in your setting?
iii)Has the HPV vaccination program elicited information from AGYWs/ their parents on their experiences with the HPV vaccine?

2.b. Cosmopolitanism

i)Is there networking happening between AGYW/ their parents regarding HPV vaccine? or with AGYW/ parents from other settings?
ii) If yes, what kind of information exchange do you have with other AGYW/ parents?

2.c. Peer Pressure

i)To what extent have other AGYW in your setting received the HPV vaccine?
ii)Has this impacted your acceptability/ your setting's uptake of the HPV vaccination program? If yes, how so?

2.d. External Policy & Incentives

i) What kind of financial or other incentives influenced your decision to receive/ allow your daughter to receive the HPV vaccine?
ii) What kind of financial or other incentives influenced the HPV vaccine uptake within your community/ in your setting?

3. Implementation context (Inner Setting)

3.a. Structural Characteristics

i)Where do most adolescents receive the HPV vaccine in your community? How is their experience in general?
ii)How did you feel about the condition of the settings (health facility, school, other) where the vaccine is provided?
iii)What kinds of infrastructure changes are needed in your setting to accommodate the delivery of HPV vaccines?

iv)How does the organization of health services in your setting affect the uptake of the HPV vaccination?

3.b. Network & Communications

i) In your opinion, what is the structure of the network of stakeholders for HPV delivery in your setting?

3.c. Implementation climate

i)In your setting was there interest or hesitation to receive the HPV vaccine? Please describe your perspective on this.

ii) [Relative Priority] To what extent does the implementation of HPV vaccination take a backseat to other high-priority initiatives going on now in your community?

3.d. Readiness for implementation

i) [Available resources] Does your setting/ community have sufficient resources to support the implementation of HPV?

ii)[Access to knowledge & Information] What kinds of information and materials on HPV vaccines have already been made available to you/ your community?

iii)[Access to knowledge & Information] Who do you ask if you have questions about the HPV vaccine or its implementation in your setting/ community?

iv) What kind of support or actions are needed to make HPV vaccine delivery successful in your community/ setting?

Probe:

➔ *Other needs your facilities/ community might have (training, materials, information, community awareness)? Are you prepared to make them available? Why?*

3.e Decision-making

i) Who is in charge of the HPV vaccination implementation at the health facility level? County-Level? Country-level? Community-level?

ii) Who influences HPV vaccine uptake in your community?

4. Characteristics of individuals

4.a. Knowledge and Belief about HPV vaccine

i)What do you know about the HPV vaccine or its implementation?

ii)What are your opinions/ perspectives toward the HPV vaccine?

iii)Do you think HPV vaccine delivery will be effective in your setting? If yes, why? If no, why not?

iv)How do you feel about the HPV vaccine being delivered to AGYW in your setting?

4.b. Self-efficacy

i) How confident are you that you/ your community will be able to use the intervention (agree to receive HPV vaccine)?
ii) How confident do you think other AGYW/ parents in your community feel about delivery of HPV vaccine?
iii) How confident do you think your other AGYW/ parents in your community feel about receiving the HPV vaccine?

<u>4.c. Individual Stage of Change</u>
i)How prepared are you to receive (your daughter to receive) the HPV vaccine?

5. Process
<u>4.a. Planning</u>
i)How was planning for implementation of the HPV vaccination carried out in your community?

<u>4.b. Engaging</u>
[Opinion Leaders/ Champions]
i)Who are the key people who have been engaged in the HPV vaccine delivery in your community/ setting?
ii) What are these key people saying about the HPV vaccine delivery?
ii) Other than formal implementation leaders, are there people in your community who are champions of HPV vaccination? How do you perceive this champion(s)? How is this champion(s) perceived in the community?

6. The Health system and policy context
<u>6.a. Health systems context</u>
i)How do you think the rollout of the HPV vaccination is going in your community/ setting?
ii) In your opinion, what knowledge and beliefs about the HPV vaccine impacted implementation? At the community and individual level?
i) Reflecting on the factors in your area, is there anything that is affecting HPV vaccine implementation? (either facilitating or hindering)

PROBE

- ➔ *Supply chain?*
- ➔ *Human resource shortages?*
- ➔ *Financial resources?*
- ➔ *Vaccine hesitancy*
- ➔ *Awareness of the cervical cancer prevention measures?*

<u>6.b. Policies and guidelines</u>
i) Are there any policies, programs, or guidelines that might affect the delivery and uptake of HPV vaccines in your community/ setting?

Probe:

- ➔ *What are they?*
- ➔ *How might they affect implementation? Scalability? Sustainability?*
- ➔ *How about government/ community directives?*

Thank you for participating in this interview. I have no further questions. Before we end the interview, is there anything you'd like to add?
[If yes, listen and continue recording, if no, stop recording and end the interview].

END TIME OF IDI: ________________________
