# Supplementary material for: HPV vaccination in Kenya: a study protocol to assess stakeholders' perspectives on implementation drivers of HPV vaccination and the acceptability of the reduced dose strategy among providers
Source: Front Health Serv. 2023 Aug 2;3:1233923. doi: 10.3389/frhs.2023.1233923 (PMC10433907; doi:10.3389/frhs.2023.1233923)
Supplement: Supplementary file 2 [file Datasheet2.pdf]

AIM 1: DECISION MAKERS & IMPLEMENTERS IN DEPTH INTERVIEW GUIDE

Instructions for the interviewer:

- Key informants are policymakers in various counties and the national Ministry of Health that are responsible for overseeing implementation of cervical cancer prevention policies and guidelines.
- Regarding our study objective, the interviewer should use, and adapt, if necessary, the lines of inquiry below to elicit participants' views on:
    - The delivery of HPV vaccines
    - The overarching health system and policy environment,
    - The implementation context (including barriers and facilitators)
    - Their capacities, and motivations

ID of data collector:<br>____
Date:<br>____
Name of Key Informant:<br>____
ID of Key Informant:<br>Eg:NBO/MOH/NIP/KI001 - Nairobi/ Ministry of Health/National Immunization Program/ Key informant #1<br>____
Job Title of Key Informant:<br>____
Level of health system where Key Informant is employed (if county, put name of county):<br>____
Number of Years Key Informant has been in that job:<br>____
Location of the interview (e.g., office, phone, clinic):<br>____
Duration of the interview (minutes):<br>____

Introduction/Opening Statement:

[READ ALOUD – INTERVIEWERS CAN PARAPHRASE]:
Thank you for taking the time to participate in this in-depth interview on the factors that may facilitate or hinder the implementation of HPV vaccine among AGYW in Kenya.

The Kenya Ministry of Health (MoH) recommendations for HPV vaccination is a two-dose schedule 0 and 6 months similar to the World Health Organization Recommendations. Kenya currently has a national HPV vaccine program based on evidence with school-based vaccination for 9-10 years which was initiated in 2019. GAVI has supported two HPV vaccination demonstration projects from 2013-2015 and 2016-present in Kitui county (outside of our proposed study areas). Both

demonstration projects used the quadrivalent HPV 16/18/6/11 vaccine in a school-based strategy that targeted girls aged 9-10 years, and the first achieved 85% coverage.
We aim to understand the key barriers and facilitators currently faced in the roll out of the HPV vaccination program. We are conducting this study to generate insights which will inform the MOH process for implementation. We are interested in your perceptions of HPV vaccine acceptability, the factors that you think may help or hinder health care workers and teams tasked with implementing HPV vaccine provision effectively and make it a sustainable component of cervical cancer prevention among AGYW in our communities.

This interview will take approximately 2h. With your permission, I would like to record the interview on a digital recorder [show the participant the digital recorder]. The recording will be kept securely and confidentially, and only used for the research purposes as described in the informed consent form [show the participant the form, which s/he has already signed]. Do I have your permission to record this interview?

INTERVIEWER SHOULD CONFIRM WITH THE KEY INFORMANT THAT S/HE HAS PERMISSION TO RECORD THE IDI ON A RECORDED DEVICE. IF RESPONDENT CONSENTS, THE INTERVIEWER CAN START RECORDING NOW. RECORD THE TIME AT WHICH THE INTERVIEW BEGINS.

START TIME OF IDI: _______________________

Interview:
0. Introduction question
Could you describe your involvement with HPV vaccination?
PROBE:

- Where they work, their position
- What is their role in this space either formal job or community leadership role
- The duration of their work in this space

1. Intervention Characteristics
<u>1.a. Intervention Source</u>

i. To what extent was the national HPV vaccination program developed externally?

<u>1.b. Evidence strength and quality</u>

i) To what extent were you provided with or made aware of the evidence supporting the introduction of the HPV vaccine?
ii) What do you and other influential stakeholders think of the HPV vaccine?
iii)What evidence is needed on the HPV vaccine to get stakeholders to support the national scale up?

PROBE

- ☐ *Quality concerns?*
- ☐ *Validity of evidence?*

☐

1.c. Relative advantage

i)Do you feel HPV vaccine is better than the existing system of screening for and identifying cervical cancer in women in Kenya?

ii) What is better HPV vaccination or cervical cancer screening?

PROBE

- ☐ *Advantage of implementing HPV vaccination vs other alternative CC preventive strategies*

1.d. Adaptability

i)How adaptable is the HPV vaccination for the different contexts in Kenya?

ii) From your perspective, what changes are needed for the HPV vaccine to be effectively rolled out in your area? (Health system for the MOH/ MOE)

iii)Who decides (or what is the process for deciding) whether changes are needed to the HPV vaccination so that it works well in your setting/ Kenya?

iv)In your opinion, what alterations should be implemented to improve uptake of HPV vaccine?

PROBE

- ☐ *What are the needed implementation adjustments and adaptations to meet local needs?*
- ☐ *How feasible are the proposed adaptations?*

1.e. Trialability

i)Do you feel the ability to implement first using a demo project was helpful? If so, why?

ii)What was the advantage of first testing the HPV vaccine implementation at a small scale?

PROBE

- ➔ The ability to test HPV vaccination roll out at small scale
- ➔ The ability to reverse course if warranted

1.f. Complexity

i) How complicated is the delivery of the HPV vaccine in Kenya?

ii) How complex was the implementation of the HPV vaccine delivery during the demo projects and what does this imply of this to the national roll out?

PROBE

- ☐ *Complicated in terms of resource allocation*
- ☐ *complicated in terms of uptake*
- ☐ *Complicated in terms of roll out and integration in the health system?*
- ☐ *Complication in terms of scale up and sustainability?*
- ☐ *Number of steps required to implement HPV vaccination*

1.g. Design Quality & Packaging

(i) What did you think about the way in which HPV vaccination demo results were presented to policymakers here in the country?
ii) How does available materials on HPV vaccination affect the national roll out in Kenya?
iii)How is public health education on the importance of the HPV vaccine undertaken?

Probe

- ➔ Was the public educated on the vaccine before its introduction into health facilities?
- ➔ How long was the public educated on the vaccine before its introduction into health facilities
- ➔ How was training done for HCWs on the importance of HPV vaccine implementation prior to the introduction of HPV vaccine in health facilities?
- ➔ How were community gatekeepers approached prior to the introduction of the HPV vaccine in health facilities?
- ➔ How is the continued community engagement post introduction of the vaccine being implemented

<u>1.h. Cost</u>

(i) What is the cost of providing full HPV vaccination to girl in Kenya (Budget Impact Analysis Aim)
ii)Are you aware of the estimated cost of implementing HPV vaccination in government health facilities/ your area?
ii) How much did it cost to implement HPV vaccination in government health facilities/ your area?
iii) Which resources were used/ are needed to roll this out?
iv) In your opinion, how should these resource needs be addressed?

Probe

- ➔ Percentage of the health budget allocated to HPV vaccine roll-out (eg; transport and storage of HPV vaccines, Staff allocations, Community education in tele and social media, Staff and personnel training)

2. Outer Setting

<u>2. a. AGYWs Needs & Resources</u>

i)In your opinion, to what extent are implementers aware of the needs and preferences of AGYWs?
ii)To what extent have AGYWs' needs been taken into consideration during the implementation of the HPV vaccination program?
iii)What barriers does AGYW face to receiving the HPV vaccine?
iv)Have you elicited information from AGYWs or heard stories about their experiences with the HPV vaccination program?

<u>2.b. Cosmopolitanism</u>

i)Did the cosmopolitanism of the specific district/ county impact implementation effectiveness? If yes, how so?

ii)Is there networking happening between the Kenyan MOH and other MOHs during the HPV roll out? or with other county managers?
iii) If yes, what kind of information exchange do you have with others outside your setting?

2.c. Peer Pressure
i)To what extent have other countries/ counties in the region implemented HPV vaccination national rollouts?
ii)Has this impacted Kenya's/ your setting's implementation of the HPV vaccination program? How so?

2.d. External Policy & Incentives
i)What kind of international policies, mandates have influenced the decision to roll out the HPV vaccine in Kenya/ your setting?
ii) What kind of financial or other incentives influenced the decision to implement the HPV vaccination in your setting?

3. Implementation context (Inner Setting)
3.a. Structural Characteristics
i)How does the infrastructure (maturity, size and coverage) of MOH/ the county/ your facility affect the roll out of the HPV vaccine?
ii)What kinds of infrastructure changes are needed to accommodate the delivery of HPV vaccines?
iii)How does the organization of health services in your setting affect the implementation of the HPV vaccination?

3.b. Network & Communications
i) What was the structure of the network of stakeholders for HPV delivery in Kenya/ your setting?
ii)Who are your influencing stakeholders? Can you describe your working relationship with influential stakeholders?
iii) When you need to get something done or to solve a problem, who are your "go-to" people?

3.c. Implementation climate
i)At the national level/ our setting was their interest or hesitation to implement HPV vaccination? Please describe your perspective on this.
ii) Were the health managers and healthcare workers in Kenya/ you're setting receptive to delivering the vaccine?
iii)[Compatibility sub-construct] How well does the HPV vaccination fit with existing work processes and practices in Kenya/your setting?

iv) [Relative Priority] To what extent might the implementation of HPV vaccination take a backseat to other high-priority initiatives going on now in Kenya or your setting?
v) [Learning sub-construct] To what extent do you/ your setting feel like you can try new things to improve HPV vaccination delivery processes?

Probe:

- ☐ *Will HPV vaccine fit in existing work processes and practices?*
- ☐ *Does the HPV vaccination align with community priorities?*

3.d. Readiness for implementation

i) [Leadership Engagement sub-construct] What has been the level of support from leaders?
ii) [Available resources] Do you/ your district/ facility have sufficient resources to support the implementation of HPV?
iii)What kinds of information and materials about the intervention have already been made available to you/ your setting?
iii) What kind of support or actions are needed to make HPV vaccine delivery successful?

Probe:

- ☐ *Are you ready to provide this support? If yes, why? If no, why not?*
- ☐ *Other needs your facilities/ community might have (training, materials, information, community awareness)? Are you prepared to make them available? Why?*

3.e Decision-making

i) Who is in charge of the HPV vaccination implementation at the health facility level? County-Level? Country-level?
ii) Who influences HPV vaccine uptake in your area/ community?

Probe

- ➔ How is the decision on the type, duration and timing of the activities about the implementation process made?

4. Process

4.a. Planning

i)How was planning for implementation of the HPV vaccination carried out at your facility, district, county, nationally?
ii) What role has the plan for implementation played during implementation?

4.b. Engaging

[Opinion Leaders/ Champions]
i)Who are the key people who have been engaged in the HPV vaccine delivery in your setting/ nationally?
ii) What are these key people saying about the HPV vaccine delivery?

iii) Other than formal implementation leaders, are there people in your setting/ nationally who are champions of HPV vaccination? How do people perceive this champion(s)?

[Formally appointed internal implementation leaders]
i) Who leads the implementation of the HPV vaccine delivery in your setting?
ii)Who else is involved with leading the implementation?

<u>4.d. Executing</u>

i)Has the HPV vaccination delivery been implemented according to the implementation plan?

<u>4.e. Reflecting and Evaluating</u>

i)How has the national rollout of HPV vaccination been monitored and evaluated?
ii)How has this been informing the national strategy?
iii)How are progress towards implementation goals being assessed?

5. The Health system and policy context

<u>5.a. Health systems context</u>

i)How do you think the rollout of the HPV vaccination is going in your area/ community/the health system?
ii) What knowledge and beliefs about the HPV vaccine impacted implementation? At facility, district, county, national, levels?
i) Reflecting on the factors in your area (health system for MOH), is there anything that is affecting HPV vaccine implementation? (Either facilitating or hindering)

PROBE

- ☐ *Supply chain?*
- ☐ *Human resource shortages?*
- ☐ *Financial resources?*
- ☐ *Vaccine hesitancy*
- ☐ *Awareness of the cervical cancer prevention measures?*

<u>5.b. Policies and guidelines</u>

i) Are there any policies, programs, or guidelines that might affect the delivery and uptake of HPV vaccines?

Probe:

- ☐ *What are they?*
- ☐ *How might they affect implementation? Scalability? Sustainability?*
- ☐ *How about government/ community directives?*

Thank you for participating in this interview. I have no further questions. Before we end the interview, is there anything you'd like to add?

[If yes, listen and continue recording, if no, stop recording and end the interview].

END TIME OF IDI: ________________________
