# Supplementary material for: HPV vaccination in Kenya: a study protocol to assess stakeholders' perspectives on implementation drivers of HPV vaccination and the acceptability of the reduced dose strategy among providers
Source: Front Health Serv. 2023 Aug 2;3:1233923. doi: 10.3389/frhs.2023.1233923 (PMC10433907; doi:10.3389/frhs.2023.1233923)
Supplement: Supplementary file 3 [file Datasheet3.pdf]

Acceptability of the Reduced Dose Strategy of HPV Vaccine

Thank you for taking the time to participate in this study and for completing this survey.

The Kenya Ministry of Health (MoH) recommendation for HPV vaccination is a two-dose schedule 0 and 6 months similar to the World Health Organization (WHO) Recommendations. Kenya currently has a national HPV vaccine program that was launched in 2019 based on evidence from a school-based vaccination for 9-10 years. GAVI has supported two HPV vaccination demonstration projects from 2013-2015 and 2016-present in Kitui county (outside of our proposed study areas). Both demonstration projects used the quadrivalent HPV 16/18/6/11 vaccine in a school-based strategy that targeted girls aged 9-10 years, and the first achieved 85% coverage.

Considering the recent endorsement of the WHO or the reduced dose strategy of the HPV vaccine for adolescent girls and young women (AGYWs), we are interested in your perceptions of how acceptable you think the reduced dose strategy is and what would make HPV vaccination a sustainable component of cervical cancer prevention among AGYW in our communities.

All your responses will be de-identified and this component of the study will not require any recording.

Do you have any questions please feel free to email or call the project PI Dr. Lynda Oluoch at lynda@pipsthika.org or 0736464299

Phone number

____
(Please note that this number will not be shared outside the KEMRI study team)

Part I: Demographics Information

Date

____

1. Modality used to complete the questionnaire

- ◯ In-person/ guided survey
- ◯ Phone
- ◯ Computer/ Laptop
- ◯ Other

(Please clarify how you are completing this survey)

1. a. Please specify

____

2. Gender

- ◯ Male
- ◯ Female

3. Age (years)

____

4. Religion

- ◯ Christian
- ◯ Muslim
- ◯ Hindu
- ◯ Other

4. a. Please specify

____

5. County	◯ Mombasa<br>◯ Kwale<br>◯ Kilifi<br>◯ Tana River<br>◯ Lamu<br>◯ Taita/Taveta<br>◯ Garissa<br>◯ Wajir<br>◯ Mandera<br>◯ Marsabit<br>◯ Isiolo<br>◯ Meru<br>◯ Tharaka-Nithi<br>◯ Embu<br>◯ Kitui<br>◯ Machakos<br>◯ Makueni<br>◯ Nyandarua<br>◯ Nyeri<br>◯ Kirinyaga<br>◯ Murang'a<br>◯ Kiambu<br>◯ Turkana<br>◯ West Pokot<br>◯ Samburu<br>◯ Trans Nzoia<br>◯ Uasin Gishu<br>◯ Elgeyo/Marakwet<br>◯ Nandi<br>◯ Baringo<br>◯ Laikipia<br>◯ Nakuru<br>◯ Narok<br>◯ Kajiado<br>◯ Kericho<br>◯ Bomet<br>◯ Kakamega<br>◯ Vihiga<br>◯ Bungoma<br>◯ Busia<br>◯ Siaya<br>◯ Kisumu<br>◯ Homa Bay<br>◯ Migori<br>◯ Kisii<br>◯ Nyamira<br>◯ Nairobi City<br>(Please pick one)
6. Name of the facility where participant works	________________________________
7. Participant Job Title	◯ Nurse<br>◯ Clinical Officer<br>◯ Medical Officer<br>◯ Pharmacist Technologist<br>◯ Pharmacist<br>◯ Hospital Director<br>◯ Other
7. a. Please specify	________________________________

8. Duration at that job

____

8. a. The duration at the job mentioned above is in

◯ Days
◯ Weeks
◯ Months
◯ Years

9. Level of the health system where the participant is employed

◯ National Referral (level 6)
◯ County Referral (level 5)
◯ County hospitals (level 4)
◯ Health center (level 3)
◯ Health dispensaries (level 2)
◯ Community Facilities (level 1)
◯ Other

9. a. Please specify

____

10. Geographic location of the facility

◯ Rural
◯ Urban
◯ Other

10. a. Please specify

____

Part II: Questions on the Acceptability of the HPV vaccine reduced dose strategy

1.General acceptability
In your opinion, how acceptable do you think it is to provide a single dose of the HPV vaccine to AGYW?

◯ Very unacceptable
◯ Unacceptable
◯ Neutral
◯ Acceptable
◯ Very acceptable

2. Burden

2.1 How much effort does it take you to provide one dose of the HPV vaccine to AGYW?

◯ No effort at all
◯ A little effort
◯ Neutral
◯ A lot of effort
◯ Huge effort

2.2. In your opinion, how much time does it/ will it take you to provide a single dose of the HPV vaccine to AGYW?

◯ No time at all
◯ Little time
◯ No opinion
◯ A lot of time
◯ A huge amount of time

2.3. In your opinion, how much resource does it/ will it take you to provide a single dose of the HPV vaccine to AGYW?

◯ No resource at all
◯ Little resource
◯ No opinion
◯ A lot of resources
◯ Huge resources

2.4 How much effort does it take to incorporate the single dose into the existing workflow at your clinic/ facility	◯ No effort at all<br>◯ A little effort<br>◯ No opinion<br>◯ A lot of effort<br>◯ Huge effort
3. Ethicality	
3.1. In your opinion, do you think there are moral or ethical consequences with offering one dose of the HPV vaccine to AGYW?	◯ Strongly disagree<br>◯ Disagree<br>◯ Neutral<br>◯ Agree<br>◯ Strongly agree
3.2. How fair is the reduced dose strategy for the AGYWs?	◯ Very unfair<br>◯ Unfair<br>◯ No opinion<br>◯ Fair<br>◯ Very fair
4. Perceived effectiveness	
4.1 To what extent do you think the HPV vaccine prevents HPV infection and cervical cancer?	◯ Not at all<br>◯ Slight protection<br>◯ Not really |No opinion<br>◯ Somehow Moderate protection<br>◯ Very much Full protection
4.2 To what extent do you think the single dose of the HPV vaccine would prevent HPV infection and cervical cancer?	◯ Not at all<br>◯ Slight protection<br>◯ Not really No opinion<br>◯ Somehow Moderate protection<br>◯ Very much Full protection
5. Self-efficacy	
5.1 How confident are you in providing (administering correctly) single doses of HPV vaccines to AGYWs?	◯ Very unconfident<br>◯ Slightly unconfident<br>◯ No opinion<br>◯ Moderately confident<br>◯ Very confident
5.2 How confident are you in explaining the benefits of the HPV vaccine to AGYWs and/ or their parents/ guardians?	◯ Very unconfident<br>◯ Slightly unconfident<br>◯ No opinion<br>◯ Moderately confident<br>◯ Very confident
5.3 How confident are you in addressing concerns that AGYWs and/ or their parents/ guardians have on the HPV vaccine?	◯ Very unconfident<br>◯ Slightly unconfident<br>◯ No opinion<br>◯ Moderately confident<br>◯ Very confident
6. In your opinion, what factors enable your ability to confidently deliver a single dose of the HPV vaccine?	________________________________<br>(What has made you/ makes you confident?)

7. What limits your confidence in delivering a single dose of the HPV vaccine?

____
(What has made you/ makes you less confident?)

8. Knowledge

8.1. How knowledgeable are you about the single-dose strategy of the HPV vaccine?

- ◯ Very unknowledgeable
- ◯ Slightly unknowledgeable
- ◯ No opinion
- ◯ Moderately knowledgeable
- ◯ Very knowledgeable

8.2. What do you know about the single-dose strategy of the HPV vaccine?

____

8.3. How did you learn about the single-dose strategy of the HPV vaccine?

____

9. What are your beliefs regarding the single-dose strategy of the HPV vaccine?

____

General Comments

Other comments?

____
